# Dependent double‐observer method reduces false‐positive errors in auditory avian survey data

**DOI:** 10.1002/eap.2026

**Published:** 2019-11-13

**Authors:** Kaitlyn M. Strickfaden, Danielle A. Fagre, Jessie D. Golding, Alan H. Harrington, Kaitlyn M. Reintsma, Jason D. Tack, Victoria J. Dreitz

**Affiliations:** ^1^ Avian Science Center and Wildlife Biology Program Department of Ecosystem and Conservation Sciences W.A. Franke College of Forestry and Conservation University of Montana 32 Campus Drive Missoula Montana 59812 USA; ^2^ National Genomics Center for Wildlife and Fish Conservation Rocky Mountain Research Station, U.S. Forest Service 800 E Beckwith Avenue Missoula Montana 59801 USA; ^3^ Animal and Rangeland Sciences Oregon State University Corvallis Oregon 97331 USA; ^4^ United States Fish and Wildlife Service Habitat and Population Evaluation Team 32 Campus Drive Missoula Montana 59812 USA

**Keywords:** abundance surveys, avian surveys, dependent double‐observer method, false positive, imperfect detection, misidentification, occupancy surveys, point counts

## Abstract

Bias introduced by detection errors is a well‐documented issue for abundance and occupancy estimates of wildlife. Detection errors bias estimates of detection and abundance or occupancy in positive and negative directions, which can produce misleading results. There have been considerable design‐ and model‐based methods to address false‐negative errors, or missed detections. However, false‐positive errors, or detections of individuals that are absent but counted as present because of misidentifications or double counts, are often assumed to not occur in ecological studies. The dependent double‐observer survey method is a design‐based approach speculated to reduce false positives because observations have the ability to be confirmed by two observers. However, whether this method reduces false positives compared to single‐observer methods has not been empirically tested. We used prairie songbirds as a model system to test if a dependent double‐observer method reduced false positives compared to a single‐observer method. We used vocalizations of ten species to create auditory simulations and used naive and expert observers to survey these simulations using single‐observer and dependent double‐observer methods. False‐positive rates were significantly lower using the dependent double‐observer survey method in both observer groups. Expert observers reported a 3.2% false‐positive rate in dependent double‐observer surveys and a 9.5% false‐positive rate in single‐observer surveys, while naive observers reported a 39.1% false‐positive rate in dependent double‐observer surveys and a 49.1% false‐positive rate in single‐observer surveys. Misidentification errors arose in all survey scenarios and almost all species combinations. However, expert observers using the dependent double‐observer method performed significantly better than other survey scenarios. Given the use of double‐observer methods and the accumulating evidence that false positives occur in many survey methods across different taxa, this study is an important step forward in acknowledging and addressing false positives.

## Introduction

Errors from imperfect detection, when left unaccounted for, are a prevalent source of bias when estimating wildlife population abundance or occupancy from field data. Observers cause two kinds of detection errors: (1) false‐negative errors, when individuals are not detected when they truly are present, and (2) false‐positive errors (“false positives”), when individuals are counted as present when they are truly absent (Royle and Link 2006, Fitzpatrick et al. 2009, Miller et al. 2011, 2012, 2015, Connors et al. 2014). There has been substantial development in methods to address false‐negative errors (Guillera‐Arroita 2017). False positives, however, have received far less attention despite recent evidence suggesting that they occur across many survey methods and taxa, including mammals such as wolves (*Canis lupis;* Miller et al. 2013) and lynx (*Lynx spp;* Molinari‐Jobin et al. 2015, Gooliaff and Hodges 2018), amphibians (Miller et al. 2012), and birds (Alldredge et al. 2008). In population‐level studies, false positives occur primarily through double counts of individuals. In community‐level studies, false positives occur through both double counts and misidentifications of individuals. We use the term “false positive” to refer only to misidentification of an individual for the remainder of this study.

**Figure 1:**
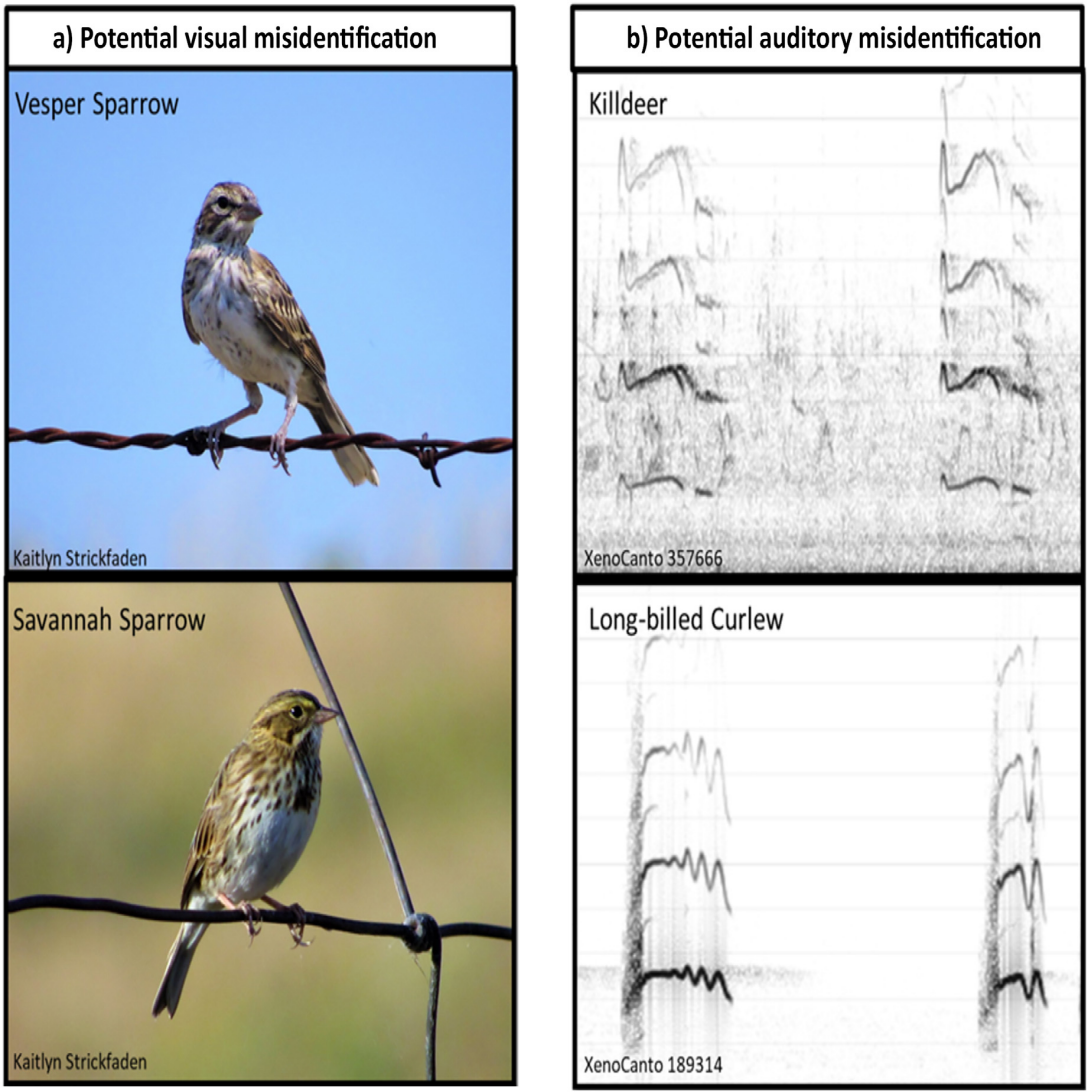
Potential sources of false‐positive error. Pictured are potential visual misidentifications between (a) Vesper Sparrow (*Pooectes graminius*) and Savannah Sparrow (*Passerculus sandwichensis*), and (b) potential auditory misidentifications illustrated through sonograms of a Killdeer (*Charadrius vociferous*) and a Long‐billed Curlew (*Numenius americanus*) call.

Several factors can affect false‐positive rates. Conditions on a survey, such as the method used or the number of species being investigated, have been shown to affect false‐positive rates. Royle and Link (2006) suggest that false positives are prevalent in multispecies surveys due to the potential for misidentification where similar species are simultaneously present (Fig. 1), although this has not been experimentally tested. A few studies have found that the rarity of a species plays a role in rates of false positives (Farmer et al. 2012, Miller et al. 2012). Observer experience and expectations can also play a role in misidentification. Farmer et al. (2012) found that experienced observers were more likely to identify common species as rare species and inexperienced observers were more likely to identify rare species as common species (Farmer et al. 2012). Last, audio frequency of avian vocalizations can also affect false‐positive rates (Miller et al. 2012).

When false positives are unaddressed, they can significantly affect inference about biological parameters. False positives increase bias and decrease precision of parameter estimates of interest (MacKenzie et al. 2002, Miller et al. 2012). Royle and Link (2006) and McClintock et al. (2010) found that false positives caused overestimation of occupancy, colonization, and local extinction probabilities in their study due to the fact that false positives were nonrandom. Miller et al. (2015) found that occupancy, colonization, and local extinction rates were biased low when detection heterogeneity caused by false positives was not taken into account. Importantly, few studies that we know of have investigated the effects of false positives on abundance estimates (but see Schaefer et al. 2015). We suspect that nonrandomness of false positives and detection heterogeneity resulting from false positives have similar but perhaps more significant effects on abundance estimates. Because misidentification of an individual causes a false positive for the incorrect species and a false‐negative error for the true species, false positives have ramifications not just on our understanding of parameters affecting a single species but on our understanding of an entire wildlife community of interest.

A variety of model‐ and design‐based solutions have been proposed to address false positives. Here we use the phrase “model‐based method” to describe a method based in statistical modeling to control for processes after data collection, while a “design‐based method” is a method based in sampling theory designed to control for processes during data collection. One model‐based option is to collect a subset of data with verifiable certainty by means such as genetic sampling or handling. These certain data are helpful in determining whether heterogeneity in occupancy or abundance in a sampling area is the result of biological differences or false positives (Miller et al. 2011), but gaining certain knowledge is not always possible. Others have suggested that instructing observers to only report observations they are certain about would eliminate false positives. While this strategy greatly increases false‐negative errors, false‐negative errors are generally easier to incorporate into models and incur less bias (Miller et al. 2012). Miller et al. (2012) found that instructing observers in this manner only slightly decreased false‐positive errors in occupancy estimates and did so very irregularly across observers and suggested against relying on this strategy to eliminate false positives. The third and most common design‐based solution to false positives has focused on observer experience with the assumption that an increase in observer experience leads to fewer false positives (Miller et al. 2015, Schaefer et al. 2015). While it is often true that experienced observers report fewer false positives, false‐positive rates reported by experienced observers are still nontrivial (Miller et al. 2012).

Independent single‐observer (ISO) methods in which a single observer counts or detects individuals over a given survey period remain the most common design for wildlife surveys (Alldredge et al. 2008, Golding and Dreitz 2016). False positives are assumed to not occur or to occur at inconsequential rates in surveys using these methods (McClintock et al. 2010, Miller et al. 2012), especially when surveys are conducted by experienced observers. ISO surveys are flexible in that the duration of sampling effort and recording method (e.g., simple counts, distance sampling) can be adjusted to meet the goals of numerous studies. However, apart from having a reliable secondary verification method (e.g., photographic, acoustic, or genetic identification), there is no way to externally verify the truth of observations beyond supposed confidence in the observer.

One promising design‐based method to reduce false positives is the dependent double‐observer (DDO) method, since varying the number of observers can have a significant effect on detection errors (Kissling and Garton 2006). Originally developed by Nichols et al. (2000) for avian point count surveys, the DDO method is based on capture–recapture removal methodology where individuals are “captured” (counted) and “removed” (not counted for the remainder of the survey) by one of the two observers in a two‐person observer team (Nichols et al. 2000, Tipton et al. 2008, 2009, Golding and Dreitz 2016). During a survey, the primary observer reports all observations to the secondary observer, who records the observations. In addition, the secondary observer records any observations that the primary observer fails to detect. The secondary observer must avoid cueing the primary observer to any missed observations by being discreet about recording observations or by faking observations (Nichols et al. 2000). Observers may also collaborate with each other in identifying an individual as long as the initial detection is attributed to the correct observer. The DDO method has been shown to be very flexible in its application and has been applied across many taxa, including songbirds (Forcey et al. 2006, Tipton 2007, Tipton et al. 2008, 2009, Leston et al. 2015; Golding and Dreitz 2016, 2017), butterflies (Henry and Anderson 2016), crocodiles (Shirley et al. 2011), and gull nests (Barbraud and Gélinaud 2005). It has been applied in arid environments including Colorado shortgrass prairie (Tipton et al. 2008, 2009), Montana sagebrush–grassland systems (Golding and Dreitz 2017), and Alberta mixed‐grass prairie (Leston et al. 2015). In addition, the DDO method lends itself well to multispecies surveys (Golding and Dreitz 2016).

The DDO method has been shown to outperform ISO methods in reducing false‐negative errors (Golding and Dreitz 2016). DDO methods can be considered design‐based and have associated model‐based solutions to accompany them, such as the multispecies dependent double‐observer abundance model, which corrects for false negatives in DDO count surveys using the double‐observer structure (Golding et al. 2017). To date, we are not aware of any model‐based mechanisms to correct for false positives in DDO surveys. Nichols et al. (2000), Golding and Dreitz (2016), and Golding et al. (2017) have suggested that the verification of individuals by two observers and the ability for observers to ask for help in identification are mechanisms by which the DDO method may reduce false positives. However, whether the method reduces false positives compared to ISO methods has not yet been experimentally tested.

In this study, we determine whether there are significant differences in the occurrence of false positives between DDO and ISO auditory survey methods based on observer experience (e.g., naive and expert). To test this, we use auditory data from 10 grassland‐ and sagebrush‐associated bird species. Bird surveys typify multispecies survey situations in which false positives are likely. Using bird species, including certain pairs that sound alike, provides a unique opportunity to explore how survey methodology, observer experience, and species identity may interact to affect false‐positive rates in complex survey situations. We predict that false positives exist in both DDO and ISO auditory surveys but that DDO surveys have lower false‐positive rates than ISO surveys. We also predict that false‐positive rates in both the DDO and ISO methods are lower for more experienced observers than for naive observers and that these rates will further vary by species. Given the potential of both approaches (i.e., survey methodology and observer experience), we feel this is an important step forward in addressing false positives in count‐based auditory wildlife surveys.

## Methods

To examine the influence of survey method and observer experience on rates of false‐positive errors, we selected 10 avian species commonly present in central Montana prairie ecosystems dominated by either grassland, sagebrush (*Artemisia spp*.), or a combination of both vegetation types. Species found in both habitat types were Brown‐headed Cowbird (*Molothrus ater*), Killdeer (*Charadrius vociferous*), Lark Bunting (*Calamospiza melanocorys*), Savannah Sparrow (*Passerculus sandwichensis*), Vesper Sparrow (*Pooectes graminius*), and Western Meadowlark (*Sturnella neglecta*). Grassland species were Long‐billed Curlew (*Numenius americanus*), Horned Lark (*Eremophila alpestris*), and McCown's Longspur (*Rhynchophanes mccownii*). Brewer's Sparrow (*Spizella breweri*) was the single sagebrush species used in the study.

### Computer generation of auditory surveys

We retrieved one vocalization for each of our study species from the Macauley Library of the Cornell Lab of Ornithology (Ithaca, New York, USA). We removed background noise from the recordings (Audacity Team 2014, Bioacoustics Research Program 2014) to avoid cuing the observers to the identity of the call and then clipped each vocalization to four seconds of audio space (hereafter, “audio clip”). We used these audio clips to generate three‐minute auditory simulations containing a random assortment of both bird vocalizations and white noise (hereafter collectively a “simulation”). Each species’ audio clip occurred in every simulation a random number of occasions between one and four. No species were overall rare compared to others, but a species could be considered rare in the context of a survey (i.e., only play once). The sequence of vocalizations and white noise was saved as a list (hereafter, “truth list”). We used these truth lists later to determine when exactly a false positive occurred in each simulation. All random compilation of audio clips into simulations was performed in R (R Core Team 2017).

### Data collection

Observers were volunteers with a wide range of bird identification experience. No training was required to take part in surveys, and experience ranged across a spectrum. Naive observers had little or no prior bird identification experience. In contrast, expert observers had multispecies avian survey experience, including observers with experience identifying the species used in this study. Each observer completed a self‐assessment to classify themselves as either “naive” or “expert” following Miller et al. (2012), who found that self‐assessment was a more accurate predictor than years of survey experience. Observers were required to participate in both DDO surveys and ISO surveys. The order of participation in each survey type was random. Observers were required to read a document explaining the ISO and DDO protocols before their first surveys, though some observers indicated that they were already familiar with these survey methods in their self‐assessments. We provided observers with a list of the 10 species along with example vocalizations for the 10 species, but we did not inform the observers of the specific vocalization for each species used in the simulations. For DDO surveys, naive observers were paired with other naive observers, and expert observers were paired with other expert observers.

We collected all data in a room with minimal background noise or distractions. We used one observer (ISO method) or two observers (DDO method) to conduct simulated surveys with protocols similar to their respective field methods. We define “simulated survey” as the process of observer(s) listening to a simulation and reporting the observed vocalizations. For ISO surveys, the observer was seated at a table with a computer at about 0.5 m distance. Simulations were played through the computer speakers at an approximate volume of 45 dB. The computer screen displayed only a timer to (1) aid the observer in keeping track of time intervals and (2) avoid visual cues for identification. The observer recorded vocalizations in 3‐s time intervals. Observers were limited to four simulated surveys per data collection period to prevent any fatigue associated with conducting surveys from influencing detection.

Methods for DDO simulated surveys were the same as what is described above with the following exceptions. An observer pair was seated 0.5 m from the computer with both observers in view of the timer on the computer screen. We randomly assigned observers the role of primary or secondary observer for their first survey. The secondary observer recorded all observations reported by the primary observer as well as any vocalizations that the primary observer failed to report. The primary observer did not have access to the recorded data, and the secondary observer was instructed to be discreet about recording additional observations during the survey. Observers switched roles as primary and secondary observer after each DDO survey. All of these procedures were put in place to keep the method as close to field implementation as possible. Observer pairs were limited to four surveys per data collection period. We paired an observer with a different partner for each data collection period.

### Data analysis

We measured rates of false positives in DDO and ISO methods by comparing the observations recorded in the simulated survey to the truth list for each simulation. We considered each audio clip to be a new individual because we had no means to indicate whether a vocalization was coming from one bird or multiple birds. In addition, the observations from the simulated surveys and the truth lists used the same time intervals, so we were able to determine which audio clips were misidentified within a survey. Because of this unique framework, the simulated surveys were more reflective of conditions in abundance surveys than occupancy surveys.

For each audio clip, we considered a 9‐s interval, which encompassed the true interval that it was played as well as the 3‐s interval on either side of the true interval, to assess each detection. The interval before accounts for observers misjudging the amount of time that passed since they heard a vocalization; vocalizations could occur consecutively depending on the randomization in survey generation and cause observers to fall behind in recording detections, causing them to misjudge when a vocalization truly occurred. The interval after accounts for delays due to human reaction time. We considered a detection correct when it was properly identified and recorded within one 3‐s interval from the true 3‐s interval (e.g., if an audio clip was played in the 1:15–1:18 time interval, the detection was considered correct if it was correctly identified in either the 1:12–1:15, 1:15–1:18, or 1:18–1:21 time interval). We classified a detection as a false positive when an audio clip was incorrectly identified or reported outside of the allowable interval.

We calculated overall and species‐specific false‐positive rates as the number of false‐positive detections divided by the total number of detections. We used logistic regression to measure differences in false‐positive rates between survey methods and experience levels and test for statistical significance (*P *<* *0.05). We inferred false‐positive rates between species based on graphical comparisons. We conducted these statistical analyses in R (R Core Team 2017).

## Results

We used a total of 12 observers, six of whom classified themselves as expert in their self‐assessments and six of whom classified themselves as naive. Expert observers completed 54 DDO surveys and 48 ISO surveys, while naive observers completed 32 DDO surveys and 43 ISO surveys for a total of 175 unique simulated surveys (Table 1). Combined, the simulations contained a total of 3,218 audio clips with a mean of 18.4 audio clips (SD = 2.7 vocalizations) and a range of 12–25 audio clips per survey. Expert observers detected 1,821 vocalizations and naive observers detected 1,290 vocalizations for a total of 3,111 detections (see Appendix S1). Logistical constraints prevented more surveys by naive observers. Nondetections, or instances when vocalizations played but were not reported by either observer, occurred a total of 107 times.

**Table 1:** Table summarizing false positive rates and standard deviations (SD) in independent single‐observer (ISO) and dependent double‐observer (DDO) simulated surveys conducted by expert and naïve observers

Survey scenario	Surveys	Detections	False positives	False positive rate	SD
Expert DDO	52	959	31	0.032	0.006
Expert ISO	48	862	82	0.095	0.010
Naive DDO	32	545	213	0.391	0.021
Naive ISO	43	745	366	0.491	0.018
Total	175	3111	692	0.222	0.007

Of the 3,111 detections, 692 were false positives, making the overall false‐positive rate 0.222 (SD = 0.007; Table 1). False‐positive rates per expert observer ranged from 0.000 to 0.132 in ISO surveys and from 0.007 to 0.066 in DDO surveys. False‐positive rates per naive observer ranged from 0.138 to 0.711 in ISO surveys and from 0.019 to 0.581 in DDO surveys.

False‐positive rates in DDO surveys were significantly lower than false‐positive rates in ISO surveys for both observer experience categories (*P *<* *0.001). Logistic regression (Table 2) indicated that observers conducting ISO surveys were 3.147 (95% CI: 2.082–4.876) times more likely to report a false positive than observers conducting DDO surveys (*P *<* *0.001). Naive observers were 19.206 (95% CI: 13.105–29.067) times more likely to report a false positive than experienced observers (*P *<* *0.001). The interaction between survey method and experience was also significant with an estimate of 0.478 (95% CI: 0.293–0.766); false‐positive rates in expert observers decreased by a greater amount when using the DDO method in comparison to the ISO method (*P *=* *0.003). In other words, the mean false‐positive rate of expert observers was 66.3% lower in DDO surveys than in ISO surveys (0.032 [SD = 0.006] and 0.095 [SD = 0.010], respectively), while the mean false‐positive rate of naive observers was 20.4% lower in DDO simulated surveys than in ISO simulated surveys (0.391 [SD = 0.021] and 0.491 [SD = 0.018], respectively).

**Table 2:** Table of logistic regression estimates, standard errors (SE), and *P* values of different survey scenarios on false positive rates in auditory surveys

Survey scenario	Estimate	SE	*P* value
Intercept	0.033	1.200	<0.001
Treatment			
Naïve observer	19.206	1.225	<0.001
ISO method	3.147	1.242	<0.001
Naïve observer × ISO method	0.478	1.277	0.003

Notes

The intercept represents expert observers using a dependent double‐observer (DDO) method. Estimates represent changes in false positive rates when naive observers conducted surveys or when surveys were conducted using an independent single‐observer (ISO) method. Logistic regression values have been transformed so that they represent the odds ratio of a false positive occurring in comparison to the intercept.

**Figure 2:**
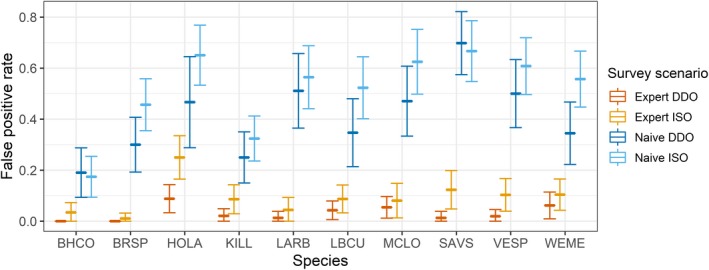
Mean false‐positive rates by species for expert and naive observers using the independent single‐observer (ISO) and dependent double‐observer (DDO) survey methods. Species are Brown‐headed Cowbird (BHCO;* Molothrus ater)*, Brewer's Sparrow (BRSP;* Spizella breweri*), Horned Lark (HOLA;* Eremophila alpestris*), Killdeer (KILL;* Charadrius vociferous*), Lark Bunting (LARB;* Calamospiza melanocorys*), Long‐billed Curlew (LBCU;* Numenius americanus*), McCown's Longspur (MCLO;* Rhyncophanes mccownii*), Savannah Sparrow (SAVS;* Passerculus sandwichensis*), Vesper Sparrow (VESP;* Pooectes graminius*), and Western Meadowlark (WEME;* Sturnella neglecta*). Error bars are 95% confidence intervals.

False‐positive rates by species differed between the two experience levels (Fig. 2). Within expert observers, the false‐positive rate of Horned Lark was higher than rates of all other study species with a false‐positive rate of 0.168 (SD = 0.026). The next closest species, Western Meadowlark, had a false‐positive rate of 0.085 (SD = 0.021). Within the naive group, false‐positive rates were generally high. The Savannah Sparrow had the highest false‐positive rate of 0.681 (SD = 0.044), and Horned Lark had a false‐positive rate of 0.591 (SD = 0.051). Brewer's Sparrow and Brown‐headed Cowbird were always detected correctly by expert observers conducting DDO surveys; otherwise, all species were misidentified at least once in all four survey scenarios. Except for naive observers detecting Brown‐headed Cowbird and Savannah Sparrow, all false‐positive rates per species were lower in DDO surveys than in ISO surveys.

**Figure 3:**
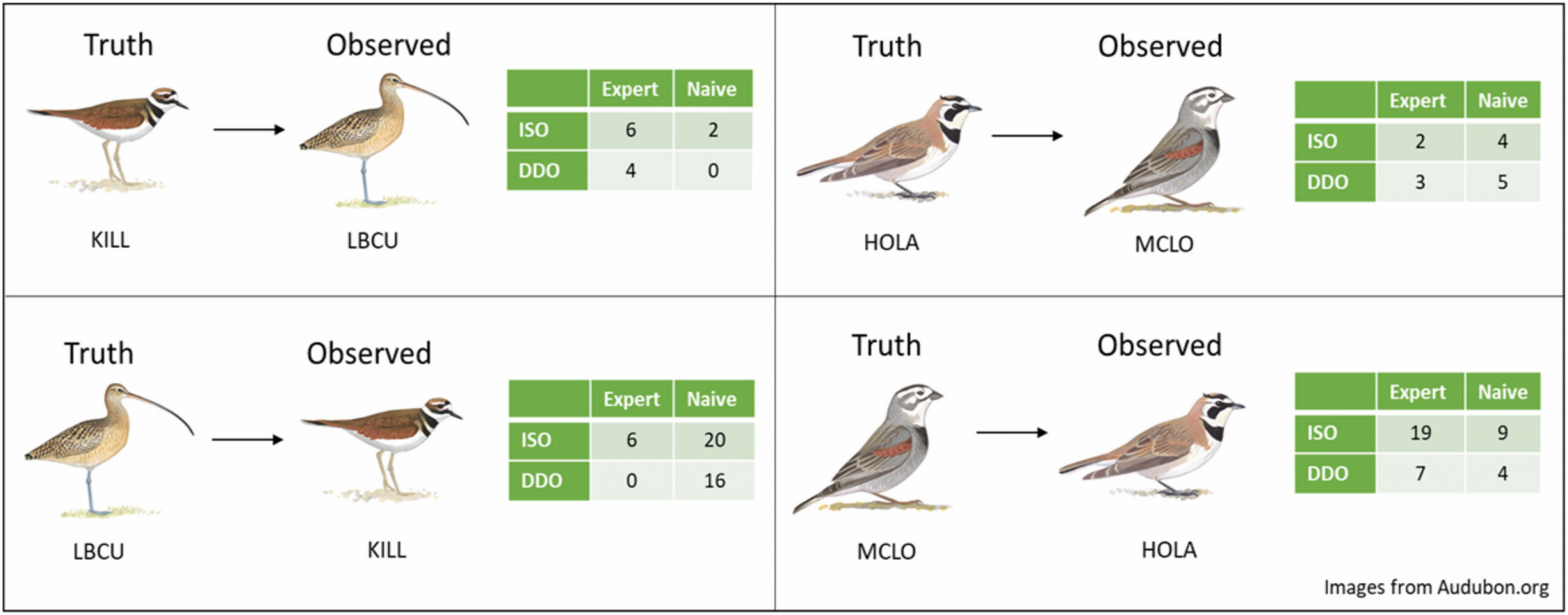
False positives in similar‐sounding pairs reported by expert and naive observers using independent single‐observer (ISO) and dependent double‐observer (DDO) survey methods. Species are Horned Lark (HOLA;* Eremophila alpestris*), Killdeer (KILL;* Charadrius vociferous*), Long‐billed Curlew (LBCU;* Numenius americanus*), and McCown's Longspur (MCLO;* Rhyncophanes mccownii*).

Accounting for both false positives and false negatives, observation totals for all study species were biased, with five species being biased low and five being biased high. McCown's Longspur observation totals were biased low by 54 observations. Killdeer, conversely, was biased high by 32 observations. Brown‐headed Cowbird was the least‐misidentified species with 30 false positives and 29 false negatives, so its observation total was biased high by only one observation. Additional patterns of bias arose in the similar‐sounding pairs (McCown's Longspur/Horned Lark and Killdeer/Long‐billed Curlew; Fig. 3). For instance, naive observers more often misidentified Long‐billed Curlew as Killdeer than they misidentified Killdeer as Long‐billed Curlew. Expert observers likewise misidentified McCown's Longspur as Horned Lark more often than they misidentified Horned Lark as McCown's Longspur.

## Discussion

False positives have received far less attention than false‐negative errors in research on wildlife population estimation despite the fact they are both capable of biasing population estimators. This study found that false positives were present in all survey scenarios. However, as predicted, false‐positive rates were significantly lower when the DDO method was used instead of the ISO method and when expert observers conducted the surveys. This indicates that a paired survey design and more experienced observers are capable of reducing, but not eliminating, false positives. The percent change in false‐positive rates between survey methods was significantly greater for expert observers (66.3%) than for naive observers (20.4%). However, naive observers reported far more false positives than expert observers, and their 20.4% reduction in false‐positive rates was the result of a 10 percentage point decrease in false‐positive rates using the DDO method, a considerable improvement over ISO methods.

Misidentifications in this study were highly variable (Fig. 2). No species observation totals were exactly equal to truth totals, and for some species, observation totals were incorrect by more than 25 observations and in both positive and negative directions. While Schaefer et al. (2015) found that abundance estimates from data collected on Kittlitz's and Marbled Murrelets (*Brachyramphus* sp.) were unbiased even with misidentifications, misidentifications occurred at approximately equal rates in their study. In this study, misidentifications in similar‐sounding pairs were often skewed in favor of one of the two species in the pair (Fig. 3). For instance, in ISO surveys by experts, Horned Larks were misidentified as McCown's Longspurs two times, but McCown's Longspurs were misidentified as Horned Larks 19 times. However, in DDO surveys by experts, Horned Larks were misidentified as McCown's Longspurs three times, while McCown's Longspurs were misidentified as Horned Larks seven times. We suspect that imbalances in misidentification such as what were seen in this study would have larger impacts on the precision and accuracy of multispecies population estimates. Based on the findings of Farmer et al. (2012), we expect that expert observers in field studies might disproportionately misidentify common species as rare species. Since occupancy estimators can become strongly biased when occupancy is low (McClintock et al. 2010, Miller et al. 2011), this tendency for experts to overcount rare species is highly problematic for biological inference in multispecies studies.

Other factors that we could not account for likely have additional impacts on detection in field studies. The most obvious of these is the visual component of detection. We did not include a visual component to detection in this study because of logistical constraints. Had we included visual cues, misidentification rates for some species could have been greatly reduced given evident differences in body shape and size. However, new misidentification patterns may have arisen between species with visual similarities. Environmental factors in the field such as distance, light, and obstructions can also influence visual detections (Diefenbach et al. 2003, Shirley et al. 2011, Henry and Anderson 2016). Another factor that may have influenced detection in this study is the standardized vocalizations we chose to include. We chose only one vocalization for each species, but all of the species have several songs and calls, some of which are less immediately identifiable to species. Similarities in vocalizations might be especially problematic in forested ecosystems where up to 95% of detections are auditory (Alldredge et al. 2008). Also, in field studies, distance and orientation of a calling bird, the volume and frequency of calls, and ambience such as noise from roads can all affect an observer's ability to detect vocalizations properly (Alldredge et al. 2008, Farmer et al. 2012). Notably, despite the removal of these potential sources of error, all false‐positive rates exceeded 3% in this study. Royle and Link (2006), McClintock et al. (2010), and Miller et al. (2011, 2015) suggested that rates as low as 1–2% can significantly bias occupancy estimates when unaccounted for, inflating occupancy estimates by as much as 50%. How false‐positive rates such as what were seen in this study would affect multispecies abundance estimates derived from field data is unknown.

One potential solution that we did not explore for reducing false‐positive rates in this study was pairing naive observers with expert observers. Fitzpatrick et al. (2009) warned against the use of observers of different experience levels in surveys. In their study, heterogeneity in detection rates biased their occupancy model; however, three observers conducted independent surveys, so the model favored the majority even if the majority was incorrect. It is possible that two dependent observers, even of different experience levels, performing the same survey would report lower bias. The DDO method allows for primary observers to ask secondary observers for identification help. This might allow a naive observer to (1) improve detection immediately by asking the more experienced observer for assistance, and (2) improve detection in future surveys by recalling learned information from past surveys (Golding et al. 2017).

The DDO method provides unique avenues for both design‐ and model‐based solutions to false positives. A secondary observer cannot correct a primary observer during a survey without violating the assumption of primary observer independence (see Nichols et al. 2000), but slight modification to the protocol could allow secondary observers to report their own identifications of individuals if they disagree with the primary. Data on disagreements could be used similarly to data with absolute certainty to calibrate population models. Assessment of detection requires either repeated sampling or sampling by distance or time at each plot (Guillera‐Arroita 2017). The DDO method allows for two observers to collect two samples of a community simultaneously, one sample containing just the primary observer's observations and the second containing both the primary and secondary observers’ observations. Occupancy estimators that included some measure of observer certainty (i.e., some observations had an unambiguous false‐positive probability of 0) have been shown to perform better (Miller et al. 2012, Chambert et al. 2015, Guillera‐Arroita 2017) because a small subset of data with an absolutely certain state of occupancy is used to calibrate the occupancy model a priori. Primary observations are often verified by the secondary observer while using the DDO method, resulting in a subset of more certain data. In addition, the multispecies dependent double‐observer abundance model developed by Golding et al. (2017) allows for random observer effects, which further mitigates issues presented by different detection rates among observers. Taken together, these design‐ and model‐based detection bias corrections unique to the DDO method allow for greater certainty in the data and in multispecies population estimates derived from these data.

## Conclusions

DDO surveys have significantly lower overall false‐positive rates than ISO surveys regardless of experience, therefore the DDO method will allow better estimates with or without directly accounting for false positives. Given the importance of observer experience in both methods, it is clear that proper training is crucial to ensuring that data collection minimizes misidentifications.

Inaccuracies in detection and the resultant biases in population estimates can misinform conclusions and lead to ineffective management practices. For avian studies that are often short‐term, accurate counts are important for detecting any changes and taking necessary action quickly. In this study, Horned Lark was vastly overcounted. In real‐world applications, if these counts are not recognized as overcounted, managers may overlook the population status of a species that is truly in decline and in need of conservation efforts. Preventing and correctly accounting for false positives where possible are both crucial for improving our understanding of wildlife communities and for effectively allocating conservation and management resources.
