# Supplementary material for: Dependent double‐observer method reduces false‐positive errors in auditory avian survey data
Source: Ecol Appl. 2019 Nov 13;30(2):e02026. doi: 10.1002/eap.2026 (PMC7078931; doi:10.1002/eap.2026)
Supplement: Supplementary file 1 [file EAP-30-e02026-s001.pdf]

Strickfaden, K.M., Fagre, D.A., Golding, J.D., Harrington, A.H., Reintsma, K.M., Tack, J.D., and Dreitz, V.J. 2019. Dependent double-observer method reduces false positive errors in auditory avian survey data. *Ecological Applications*.

Data S1

Observations from auditory avian survey simulations.

Authors

Kaitlyn M. Strickfaden
Avian Science Center, W.A. Franke College of Forestry and Conservation, University of Montana, 32 Campus Drive, Missoula, MT 59812, USA.
Kaitlyn.strickfaden@gmail.com

Danielle A. Fagre
Avian Science Center, W.A. Franke College of Forestry and Conservation, University of Montana, 32 Campus Drive, Missoula, MT 59812, USA.
Danielle.fagre@gmail.com

Jessie D. Golding
Avian Science Center, W.A. Franke College of Forestry and Conservation, University of Montana, 32 Campus Drive, Missoula, MT 59812, USA.
National Genomics Center for Fish and Wildlife Conservation, Rocky Mountain Research Station, U.S. Forest Service, 800 E Beckwith Avenue, Missoula, MT 59812, USA.
Jessie.golding@umontana.edu

Alan H. Harrington
Avian Science Center, W.A. Franke College of Forestry and Conservation, University of Montana, 32 Campus Drive, Missoula, MT 59812, USA.
Animal and Rangeland Sciences, Oregon State University, Corvallis, OR 97331, USA.
Alan.harrington001@gmail.com

Kaitlyn M. Reintsma
Avian Science Center, W.A. Franke College of Forestry and Conservation, University of Montana, 32 Campus Drive, Missoula, MT 59812, USA.
Kaitlyn.reintsma@umontana.edu
Jason D. Tack

Avian Science Center, W.A. Franke College of Forestry and Conservation, University of Montana, 32 Campus Drive, Missoula, MT 59812, USA.
United States Fish and Wildlife Service, Habitat and Population Evaluation Team, 32 Campus Drive, Missoula, MT 59812, USA.
Jason_tack@fws.gov

Victoria J. Dreitz
Avian Science Center, W.A. Franke College of Forestry and Conservation, University of Montana, 32 Campus Drive, Missoula, MT 59812, USA.
Victoria.dreitz@umontana.edu

File List (files found within DataS1.zip):

`Strickfaden_et_al_Dataset.csv`

Description

Observation from known-truth auditory avian simulation surveys conducted by naïve (N) and expert (E) observers using either the Independent Single-Observer (ISO) or Dependent Double-Observer (DDO) survey method. Observations are designated as either a True Positive detection (TP) or a False Positive detection (FP). Species reported in the dataset are BHCO – Brown-headed Cowbird (*Molothrus ater*), BRSP – Brewer's Sparrow (*Spizella breweri*), HOLA – Horned Lark (*Eremophila alpestris*), KILL – Killdeer (*Charadrius vociferous*), LARB – Lark Bunting (*Calamospiza melanocorys*), LBCU – Long-billed Curlew (*Numenius americanus*), MCLO – McCown's Longspur (*Rhyncophanes mccownii*), SAVS – Savannah Sparrow (*Passerculus sandwichensis*), VESP – Vesper Sparrow (*Pooectes gramineus*), and WEME – Western Meadowlark (*Sturnella neglecta*).
